# Isolated hallucination is less predictive than thought disorder in psychosis: Insight from a longitudinal study in a clinical population at high risk for psychosis

**DOI:** 10.1038/s41598-018-32215-6

**Published:** 2018-09-18

**Authors:** TianHong Zhang, LiHua Xu, YingYing Tang, HuiRu Cui, YanYan Wei, XiaoChen Tang, Qiang Hu, Yan Wang, YiKang Zhu, LiJuan Jiang, Li Hui, XiaoHua Liu, ChunBo Li, JiJun Wang

**Affiliations:** 1 Shanghai Mental Health Center, Shanghai Jiaotong University School of Medicine, Shanghai Key Laboratory of Psychotic Disorders, Shanghai, 200030 P. R. China; 2 Institute of Mental Health, The Affiliated Guangji Hospital of Soochow Unversity, Soochow Unversity, 215137 Suzhou, P. R. China; 3 CAS Center for Excellence in Brain Science and Intelligence Technology (CEBSIT), Chinese Academy of Science, Shanghai, P. R. China; 4 Bio-X Institutes, Key Laboratory for the Genetics of Developmental and Neuropsychiatric Disorders (Ministry of Education), Shanghai, China

## Abstract

Perceptual abnormalities (PAs) such as auditory hallucinations are one of the most common symptoms of psychotic patients. However, it remains unclear whether symptoms of sub-clinical PAs also play a key role in predicting psychosis. In an ongoing prospective follow-up study of individuals at a clinical high risk (CHR) of psychosis, we evaluated the potential of first-time experience of PAs and/or thought content disorders (TCDs) to predict psychosis. Conversion to psychosis was the major focus of this follow-up study. A total of 511 CHRs were recruited, of whom 443 (86.7%) completed the clinical follow-up of at least 6 months and up to 2 years. CHRs were divided into four groups according to the presence of PAs and/or TCDs. At the follow-up endpoint, 39 (19.9%) CHRs in the “TCDs-only” group, 2 (8.3%) in the “PAs-only” group, 45 (17.0%) in the “TCDs-and-PAs” group, and 1 (3.8%) in the “None” group converted to psychosis. Survival analysis revealed a higher conversion rate in CHRs with TCDs compared with those with PAs only. CHRs with isolated PAs had shown a higher level of dysphoric mood at baseline compared with those with TCDs. About 89% TCDs contents were related with their experienced PAs. Compared with TCDs, the isolated PAs are not strongly associated with increased susceptibility to psychosis.

## Introduction

Perceptual abnormalities (PAs), including auditory hallucinations, and thought content disorders (TCDs), including delusions, are the two most common symptoms that occur during the course of psychosis^1^; in the early psychosis stage, these symptoms are identified as clinical high risk (CHR)^2,3^. Unlike TCDs, which appear insidiously, PAs are more easily identified both by professionals and non-professional, and are often deemed as “insanity” resulting in extreme fear, misery, and stigma for those experiencing a hallucinatory state for the first time. However, none of the previous studies^4,5^ reported PAs as a valuable predictive factor in psychosis risk calculations. Currently, misconceptions exist in the public attitude and psychiatric practice toward hallucinations because there is no evidence to challenge the conventional opinion that confuses hallucinations with psychosis. Because of these potentials, we hypothesized that the isolated PAs are less specific early symptoms than TCDs in predicting conversion to full psychosis. This large-scale follow-up study was designed to evaluate the potential of first-time experience of TCDs and/or PAs to predict psychosis.

## Method

### Baseline assessment

The current study was conducted at the Shanghai Mental Health Centre (SMHC). The study was conducted following the tenets of the Helsinki Declaration and approved by the Research Ethics Committee of the SMHC in 2011. All participants gave written informed consent at the recruitment stage of the study. In total, 511 participants with CHR (CHRs) were consecutively recruited based on a face-to-face interview during follow-up every 6 months without any extra intervention programs. All participants provided written informed consent. Those younger than 18 years of age provided assent and were signed up for the study by their parents, who provided consent. The sampling approach, interviews, and follow-up methods have been published extensively elsewhere^6,7^. Inclusion criteria were: (i) age of 14–45 years; (ii) individuals younger than 18 years who were accompanied by either their parent or legal guardian; (iii) capacity to provide informed consent; and (iv) completed at least 6 years of primary education. Exclusion criteria were: (i) severe somatic diseases, such as pneumonia, cancer, or heart failure; (ii) mental retardation; or (iii) dementia. Participants were a consecutive series from the psychological counseling service setting according to the hospital register form.

This observational study sample was taken from the Shanghai Psychotherapy and Psychological Counseling Center (SPCC) at SMHC, which is China’s largest outpatient medication-management and psychotherapy providing mental health clinic. All participants are making their first visit for mental health service and psychotropically naïve when they enter the study and are assessed clinically. Participants are not treated in the study, but receive treatment as usual by their community psychiatrist after their baseline assessment, as needed.

The Structured Interview for Prodromal Symptoms (SIPS) and Scale of Prodromal Syndromes (SOPS)^8^ were used to determine the CHR status. The SOPS consisted of 19 items (scale range from 0 to 6) assessing four symptom domains: Positive symptoms (Scales P1–P5), Negative symptoms (Scales N1–N6), Disorganized symptoms (Scales D1–D4), and General symptoms (Scales G1–G4). The types and severity of the clinical symptoms (TCDs and PAs) were assessed by the psychiatrists at each visit using a scale based on the SOPS. TCDs was defined as CHRs who received a rating level of ‘3’ or higher on any of the P1–P3 scales of the SOPS (P1: Unusual Thought Content, P2: Suspiciousness, and P3: Grandiosity). PAs was defined as CHRs who received a rating level of ‘3’ or higher on the P4 scale of the SOPS (Perceptual Abnormalities). The dysphoric mood was measured using general symptoms (item 2) of the SOPS, which evaluates the symptom severity across diminished interest in pleasurable activities, sleep problems, difficulty concentrating, suicidal thoughts, anxiety, panic, irritability, hostility, tension, and unstable mood etc. from 0 (absent) to 6 (extreme). There were inquiries during the interview for those CHRs with PAs and TCDs as to whether the contents of PAs were related to TCDs. Details of the symptoms identified through the SIPS interview were recorded in vignettes for all CHRs.

The English version of SIPS/SOPS has demonstrated acceptable inter-rater reliability and predictive validity^8,9^. With written permission, the SIPS/SOPS (Version 5) was translated into Chinese according to the strict international translation standard^10^ by our team. Preliminary SIPS data collected at SMHC^10^ revealed good inter-rater reliability (r = 0.96, p < 0.01 on the SOPS score). Expressed as the kappa value, the agreement rate between the two psychiatrists was 0.81. The Cronbach’s α for all SOPS items was 0.71, and the total SOPS score correlated significantly with the Chinese Positive and Negative Syndrome Scale (PANSS) total score (r = 0.63, p < 0.01).

### Follow-up assessment

Up to April 2017, 443 (86.7%) CHRs completed at least a 6-month follow-up with a mean follow-up time of 13.7 (standard deviation [SD] = 7.1) months. The major focus of this follow-up study was conversion to psychosis. Conversion was determined using the criteria of Presence of Psychotic Symptoms (POPS; in SIPS/SOPS)^11^. Participants had to demonstrate at least one psychotic level symptom and a rate of “6” on the five positive symptoms with either sufficient frequency (average, 4 days/week), duration (1 month or 1 hour/day), or urgency (disorganizing or dangerous symptoms). Both the patients and their caregivers had been told that they could contact the interviewer and study clinicians anytime for questions and reporting on the patients’ medical conditions during the subsequent 2 years. Except for those who did not desire any further contact, the CHRs were re-assessed every 6 months by face-to-face interviews or by telephone using SIPS/SOPS.

### Statistical analysis

The initial objective of this analysis was to compare the outcomes of CHRs with PAs and/or TCDs. Therefore, based on the identified symptoms, CHRs were divided into four groups based of occurrence of TCDs and Pas, and consisted of “TCDs-only,” “PAs-only,” “TCDs-and-PAs,” “none.” Baseline characteristics of CHRs were summarized using descriptive statistics. Differences in the CHRs characteristics between TCDs and/or PAs cohorts were compared using chi-square tests for categorical data and non-parametric Kruskal-Wallis tests for numerical variables. Independent-samples t-tests were used to compare between groups, as applicable. Significance was determined by *p* < 0.05 (two-tailed). Kaplan-Meier survival curves of time to conversion were plotted for each TCDs and/or PAs cohort. The overall difference between survival curves was compared using a log-rank test. We further evaluated the predictive values (e.g., sensitivity and specificity) of the severity of PAs/TCDs symptoms, which was derived from the P1–P3 scales of the SOPS(TCDs) and the P4 scale of the SOPS(PAs). The score ranges of TCDs (0–18) and Pas (0–6) were dissimilar. We, therefore, transformed the raw scored of TCDs and PAs into standardized z-scores. Receiver operating characteristic (ROC) analysis was used to test, whether the severity of PAs/TCDs symptoms (z-scores) allows distinguishing between converters and non-converters.

## Results

Of the 511 CHRs (mean age = 20.6 years, SD = 6.2, 53% female) recruited within the study period, 196 were “TCDs-only,” 24 “PAs-only,” 265 “TCDs-and-PAs,” 26 “None.” Table 1 summarizes the baseline characteristics of CHRs. The score of disorganized symptoms was significantly higher in those with TCDs-only, compared with those with PAs-only.

**Table 1:** Baseline demographic and clinical variables, comparing participants with clinical high risk (CHRs) with perceptual abnormalities (PAs) only or thought content disorders (TCDs) only.

Variables	Total Sample	TCDs-only	TCDs-and-PAs	PAs- only	None	TCDs-only *VS*. PAs-only	
						*t/χ* ^2^	*p value*
Cases (*n*)	511	196	265	24	26	—	—
Age (years), (*Mean* [*standard deviation*, *SD*])	20.6 (6.2)	21.6 (6.1)	19.0 (5.4)	21.9 (8.4)	28.4 (6.3)	−0.240	0.810
Male (*n* [%])	241 (47.2)	112 (57.1)	104 (39.2)	10 (41.7)	15 (57.7)	2.073	0.150
Education (years), (*Mean* [*SD*])	11.2 (3.0)	11.8 (3.2)	10.6 (2.7)	11.5 (2.9)	13.7 (3.3)	0.370	0.218
Marital status-Single/separated/divorced, (*n* [%])	451 (88.3)	164 (83.7)	255 (96.2)	18 (75.0)	14 (53.8)	1.126	0.289
**SIPS/SOPS (Structured Interview for Prodromal Symptoms and the Scale of Prodromal Syndromes)**							
Negative symptoms, (*Mean* [*SD*])	11.6 (5.8)	11.4 (6.1)	12.0 (5.5)	9.9 (6.4)	10.3 (5.3)	1.168	0.244
Disorganized symptoms, (*Mean* [*SD*])	5.7 (3.2)	5.6 (3.0)	6.3 (3.2)	3.0 (1.7)	2.6 (1.7)	4.032	**<0**.**001**
General symptoms, (*Mean* [*SD*])	9.0 (3.2)	8.2 (3.3)	9.4 (2.9)	8.7 (3.9)	10.6 (3.1)	−0.724	0.470
Current GAF, (*Mean* [*SD*])	55.7 (7.5)	56.7 (7.8)	54.6 (7.3)	59.8 (8.2)	54.3 (3.4)	−1.790	0.075
Drop GAF, (*Mean* [*SD*])	23.1 (7.4)	21.7 (7.3)	24.2 (7.4)	19.0 (8.2)	26.6 (3.7)	1.689	0.093

The global assessment of function (GAF) was used as a measure of the global psychological, social, and occupational functioning of the CHRs in the SIPS/SOPS interview. Drop GAF, current GAF score from highest in past year.

The scores of positive symptoms in SOPS were used for grouping CHRs. Negative/Disorganized/General symptoms are also rated on a SOPS scale that ranges from 0 (Absent) to 6 (Extreme). Negative symptoms were as follows: N1, Social Anhedonia; N2, Avolition; N3, Expression of Emotion; N4, Experience of Emotions and Self; N5, Ideational Richness; and N6, Occupational Functioning. Disorganized symptoms were as follows: D1, Odd Behavior or Appearance; D2, Bizarre Thinking; D3, Trouble with Focus and Attention; D4, Personal Hygiene. General symptoms were as follows: G1, Sleep Disturbance; G2, Dysphoric Mood; G3, Motor Disturbances; and G4, Impaired Tolerance to Normal Stress.

Of the 511 CHRs analyzed, 68(13.3%) were lost to follow-up, while 73, 206, 98, and 66 CHRs reached the 6-, 12-, 18-, 24-month follow-up, respectively. At the follow-up endpoint, 39 (19.9%) CHRs in the “TCDs-only” group, 2 (8.3%) in the “PAs-only” group, 45 (17.0%) in the “TCDs-and-PAs” group, and 1 (3.8%) in the “None” group converted to psychosis (Table 2).

**Table 2:** Characteristics of participants with clinical high risk (CHRs)–comparison between converters and non-converters of psychosis.

Variables	TCDs-only		TCDs-and-PAs		PAs- only		None	
	Converters	Non-converters	Converters	Non-converters	Converters (Case 1,2)	Non-converters	Converters (Case 1)	Non-converters
Cases (*n*)	39	129	45	192	2	20	1	**15**
Age (years), (*Mean* [*SD*])	20.7 (5.7)	21.5 (5.9)	20.0 (5.6)	18.5 (5.1)	16, 17	23 (8.8)	18	**28**.**5 (6**.**5)**
Male (*n* [%])	23 (59.0)	72 (55.8)	**24** (**53**.**3**)	**70** (**36**.**5**)*	1 (50)	8 (40.0)	1 (100)	**8 (53**.**3)**
Education (years), (*Mean* [*SD*])	11.5 (2.9)	11.8 (3.3)	10.8 (2.6)	10.5 (2.7)	7, 9	11.9 (2.9)	10	**13**.**3 (3**.**0)**
Single/separated/divorced, (*n* [%])	36 (92.3)	108 (83.7)	42 (93.3)	188 (97.9)	2 (100)	14 (70.0)	1 (100)	**9 (60**.**0)**
Negative symptoms, (*Mean* [*SD*])	**13**.**9** (**6**.**5**)	**10**.**8** (**5**.**6**)**	**13**.**6** (**6**.**0**)	**11**.**7** (**5**.**5**)*	10, 10	9.9 (7.0)	17	**12**.**1 (4**.**8)**
Disorganized symptoms, (*Mean* [*SD*])	5.9 (2.9)	5.5 (3.0)	6.8 (3.0)	6.2 (3.2)	1, 4	3.1 (1.7)	3	**2**.**9 (1**.**9)**
General symptoms, [*Mean* (*SD*)]	7.6 (3.3)	8.6 (3.2)	9.1 (2.6)	9.5 (3.0)	12, 3	9.0 (3.9)	14	**10**.**4 (3**.**6)**
Current GAF, (*Mean* [*SD*])	**54**.**0** (**6**.**0**)	**56**.**9** (**7**.**5**)*	52.6 (6.0)	54.9 (7.5)	55, 58	59.5 (8.8)	52	**53**.**6 (3**.**3)**
Drop GAF, (*Mean* [*SD*])	24.3 (6.3)	21.2 (7.1)*	27.2 (6.2)	23.7 (7.2)**	**20**, **22**	**19**.**6 (8**.**6)**	**28**	**26**.**6 (3**.**9)**

GAF, The global assessment of function. *p < 0.05; **p < 0.01.

**Figure 1:**
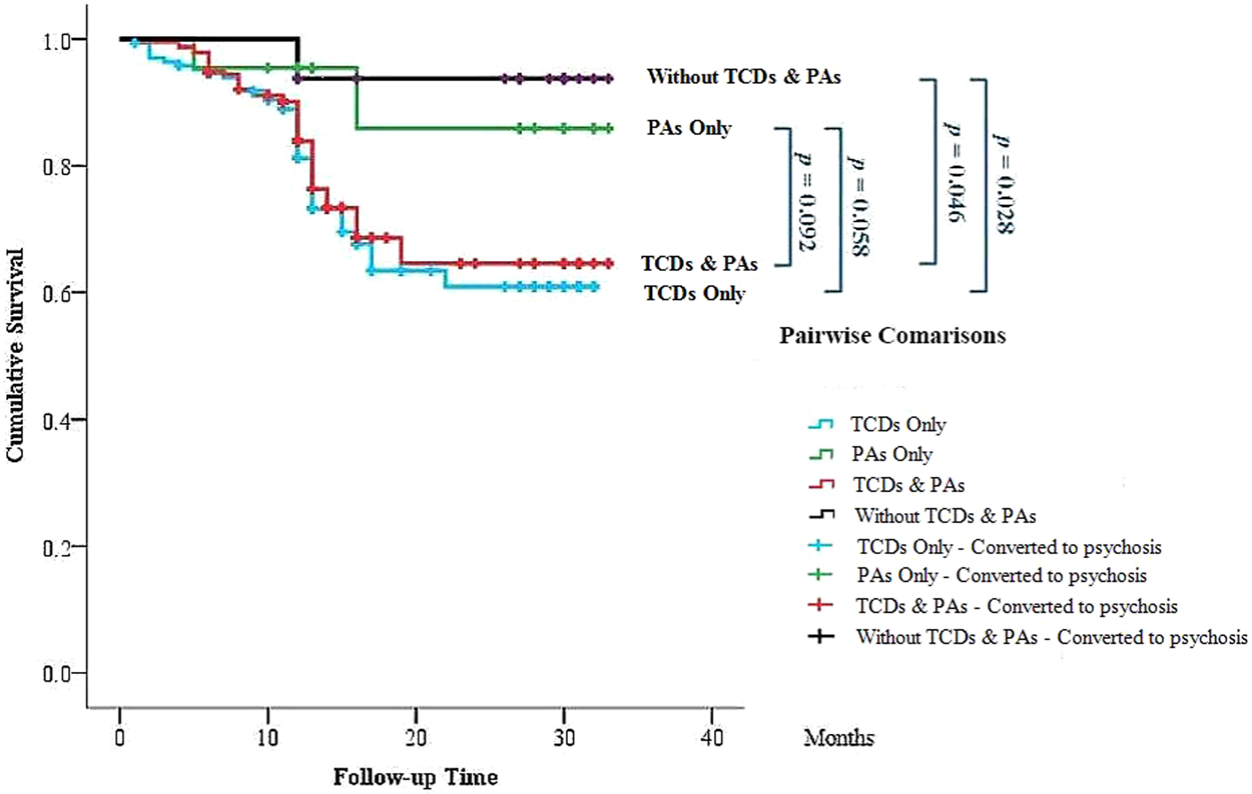
Survival curves for conversion to psychosis among clinical high risk (CHR) with or without perceptual abnormalities (PAs)/thought content disorders (TCDs). The difference between survival curves was compared using a log-rank test.

To test the main hypothesis, Kaplan-Meyer survival curves were constructed for 443 CHRs (87 converters and 356 non-converters) separated by PAs and/or TCDs symptoms. In Fig. 1A, survival curves showed that the conversion rate was higher in CHRs with TCDs compared with those with PAs only.

**Figure 2:**
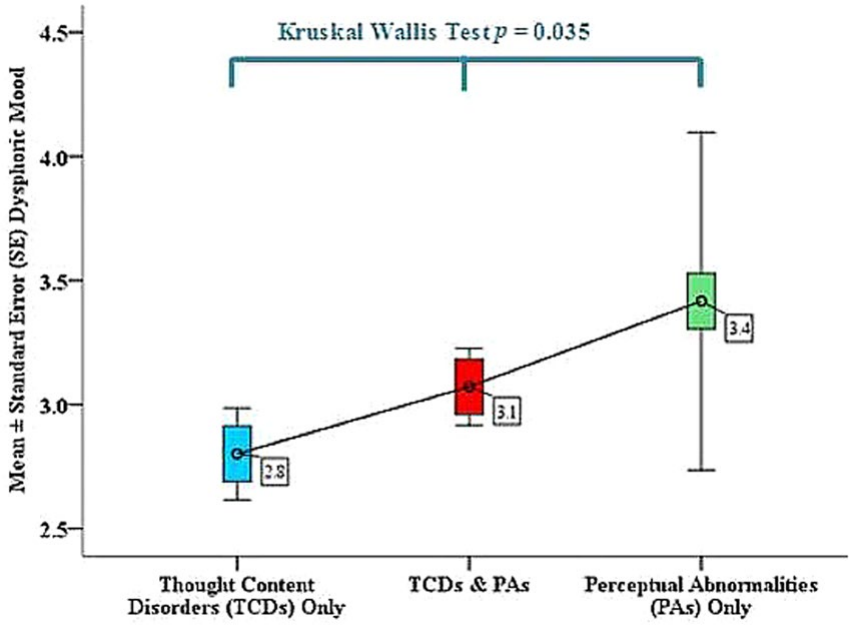
Dysphoric mood symptoms among CHRs with or without perceptual abnormalities (PAs)/thought content disorders (TCDs).

However, at the baseline, CHRs with isolated PAs (most of whom did not convert to psychosis) had shown a higher level of dysphoric mood compared with those with TCDs (Fig. 2). The next step was to examine whether the contents of TCDs could be related to the experienced PAs. The vignettes of CHR participants with TCDs and PAs symptoms were included in the analysis. As expected, the majority of TCDs contents (211/237, 89.0%) reported by CHRs were related with their experienced PAs. For example, CHRs reported TCDs as feeling as if people around them were judging them in a negative way, and PAs as hearing negative comments from others. Another example, TCDs as thinking that maybe other people could “read” their mind, and PAs as hearing their thoughts spoken aloud outside of their head.

We further investigated whether the severity of PAs or TCDs symptoms could discriminate converters from non-converters. When conversion to psychosis is the principal endpoint, the ROC analysis resulted in an area under the curve (AUC) of 0.522 (*p* = 0.471) for the severity of PAs symptoms. However, the AUC increased to 0.648 (*p* < 0.001) for the severity of TCDs symptoms.

## Discussion

Although auditory hallucination is a common symptom in patients with psychosis, fewer CHRs with isolated PAs converted to psychosis compared with those with TCDs in this study. This suggests that PAs are not specific predictors for the onset of psychosis. Nevertheless, CHRs are under enormous pressure to deal with recent-onset PAs^12^, which may not be a precursor for psychosis. Mertin and O’Brien^13^, in their study of non-psychotic children, confirmed auditory hallucinations is strongly associated with the presence of high levels of emotional distress. Furthermore, TCDs may arise in an attempt to explain PAs^14^. Once delusional interpretations (i.e., TCDs) of PAs occur, the conversion risk would increase accordingly; this may explain the approximate 90% TCDs contents characterized by having contextual PAs.

There is a dearth of reports on the patterns of emergence of PAs and TCDs symptoms, and it remains unclear which of the two, PAs or TCDs, occur first^15^. Our results suggested that the pattern of emergence of PAs and TCDs may be associated with varying levels of risk for psychosis. Isolated PAs symptoms associated with lower risk for psychosis compared to TCDs symptoms, which indicated PAs, may not reflect the emergence of a core psychosis. A recent study from Schimmelmann *et al*.^16^ reported that perceptive attenuated psychotic symptoms (APS) were generally less related to functional impairment, however, non-perceptive APS were related to low functioning. Many studies also reported that isolated hallucinatory experiences exist within the general population^17,18^. In a previous study^19^ of 3870 general children in 7- and 8-year-olds, the 1-year prevalence of auditory vocal hallucinations was 9%, and the persistence of hallucinations in later 5 or 11 years is not uncommon^20,21^. Therefore, our data further confirmed that, unlike TCDs that are associated with the emergence of psychotic process, isolated PAs may play a role of “clinical noise” or normal variations among the general population^22^. Actually, CHR individuals with isolated PAs are more likely to have a mood or anxiety disorder than a psychotic illness during the follow-up^23^.

Interestingly, the disorganized symptoms reported by CHRs with isolated PAs are more severe than those with TCDs. Of the individual items of disorganized symptoms, D1-Odd Behavior of Appearance, D2-Bizarre Thinking, D3-Trouble with Focus and Attention and D4-Impairment in Personal Hygiene, the D2 item is partially overlapped with TCDs. Thus, we conducted re-analysis by excluding the D2 from the disorganized symptoms scores, and found that no significant difference among groups.

Thus, the current study reveals that isolated PAs is not an adequate proxy for CHR diagnosis or be treated as prodromal psychotic symptoms. Particularly, it should be extreme caution for those CHR subjects with PAs are identified through self‐rating questionnaires^24^. Previous studies have shown that questionnaire method is very likely to overestimate the presence of psychotic‐like experiences in comparison to face-to-face interview method^25,26^. The PAs may be more suited to be treated as “a transdiagnostic dimension of psychopathology” and “a marker for the severity of non‐psychotic states”^27^, than prodromal psychosis.

Several limitations must be considered when interpreting the present findings. First, this study was an observational study and CHRs were not surveyed naturally. The various medications that the CHRs were taking with different compliance during the follow-up periods may have also confounded the results. Nevertheless, this is also a strength point in the study because it reflects better the real world. Second, CHRs in this study were outpatients of the psychological counseling clinic in Shanghai, thus limiting the generalization of our findings to the general population. Third, the sample of CHRs with PAs-only was relatively small, making the conclusion less convincing. Many studies reported the high prevalence rates of non-psychotic hallucination among general population especially in youth. However, to the best of our knowledge, this is the only study to include in the analysis a clinical population of CHRs with isolated PAs symptoms. Fourth, the age of eligible sample in current study was slightly biased against 14–21 years old, this bias may influence our findings. The age-effect^28^ on PAs in CHR sample had been noticed, especially in under-16-year-olds^29^. Further studies of CHR youths in comparison to adults group are needed, in order to avoid misinterpretation of psychopathological nature in child/adolescents^16^. Finally, this sample received a naturalistic treatment, 56% of the sample who were taking antipsychotics or antidepressants, and this factor may have confounded the outcome, limiting the generality of our findings to CHR subjects in general population who have not taken any psychiatric treatment.

Despite these limitations, our findings offered valuable insights into the necessity to change the public prejudices about sub-clinical hallucinations. Obviously, objective acceptance for PAs in high-risk populations may offer advantages over unnecessary over-reactions (excessive worry, anxiety, and depression). Thus, preventing secondary TCDs is likely to become of primary importance, such as suspiciousness and reference ideas that can be induced by auditory hallucination. Future efforts should develop effective psychotherapy focusing on isolating the PAs from TCDs in persons with CHR. This may help diminish the stigma and the prejudices of the public in the face of individuals with PAs.
